# Resting-state frontal theta/beta ratio and psychological symptoms in college students: an EEG study

**DOI:** 10.3389/fpsyt.2026.1834200

**Published:** 2026-09-18

**Authors:** Duo Mi, Yuanyuan Li, Yan Zhu, Zhaoxin Guo, Lun Zhao

**Affiliations:** 1 Mental Health Guidance Center, Ningbo University, Ningbo, China; 2 Institute of Psycho-Brain Health, Chutzpah Digital Intelligence Technology (Zhejiang) Co. Ltd., Ningbo, China

**Keywords:** Cognitive control, college students, mental health, psychological symptoms, resting-state EEG, symptom checklist-90, theta–beta ratio

## Abstract

**Background:**

Mental health problems are increasingly prevalent among college students and represent an important public health concern. Neurophysiological indicators derived from electroencephalography (EEG) may provide objective markers for identifying psychological vulnerability. The theta/beta ratio (TBR) has been widely associated with attentional regulation and cognitive control; however, its relationship with multidimensional psychological symptoms in non-clinical populations remains insufficiently understood.

**Methods:**

Resting-state EEG was recorded from 222 undergraduate students aged 18–25 years during an eyes-closed condition. Psychological symptoms were assessed using the Symptom Checklist-90. Theta (4–7 Hz) and beta (14–30 Hz) power were extracted from frontal electrodes (F3 and F4), and the theta/beta ratio was calculated. Group comparisons and correlation analyses were conducted to examine associations between EEG measures and psychological symptom severity.

**Results:**

Students with higher levels of psychological symptoms showed significantly greater frontal theta and beta power compared with those reporting low-score levels. In addition, the theta/beta ratio was significantly higher in students with greater psychological symptom severity and was positively correlated with the total SCL-90 score as well as several symptom dimensions.

**Conclusions:**

These findings suggest that resting-state frontal TBR is associated with psychological symptom severity in college students. The theta/beta ratio may serve as a potential neurophysiological candidate correlate of mental health vulnerability in non-clinical populations and provide a reference for objective auxiliary assessment of mental health in university populations.

## Introduction

Mental health problems among university students have become an increasingly significant public health concern worldwide. The transition to university life often involves substantial academic, social, and psychological challenges that may increase vulnerability to emotional distress. Students frequently encounter academic pressure, financial concerns, changing social networks, and uncertainty regarding career development, all of which may contribute to psychological symptoms during emerging adulthood (1, 2). Epidemiological research indicates that the prevalence of mental health problems among college students has increased substantially over the past decade, highlighting the importance of identifying risk factors and early indicators of psychological vulnerability (3, 4). These psychological difficulties can negatively affect academic performance, interpersonal functioning, and long-term well-being (5).

Psychological distress in university populations is typically assessed through self-report questionnaires or clinical interviews. One of the most widely used instruments for assessing multidimensional psychological symptoms is the Symptom Checklist-90 (SCL-90), which evaluates a broad range of psychological problems including depression, anxiety, somatization, hostility, interpersonal sensitivity, and obsessive–compulsive symptoms (6). The SCL-90 has demonstrated strong reliability and validity across different cultural and clinical contexts and is frequently used in studies investigating mental health among college students (7). Although self-report scales provide valuable insight into subjective experiences of distress, they may be influenced by reporting biases and individual differences in emotional awareness (8). Therefore, the integration of objective neurophysiological indicators may provide complementary information regarding the neural processes underlying psychological symptoms.

Electroencephalography (EEG) is a widely used non-invasive technique that measures electrical activity of the brain with high temporal resolution. Resting-state EEG, in particular, allows researchers to examine spontaneous neural oscillations without the influence of task-related cognitive demands (9). Neural oscillations are typically categorized into several frequency bands, including delta, theta, alpha, beta, and gamma rhythms, each of which is associated with specific neural functions and cognitive processes (10). Increasing evidence suggests that abnormalities in EEG oscillatory activity are associated with a variety of psychiatric and psychological conditions, including depression, anxiety disorders, and attention-related disorders (11, 12).

Among these oscillatory rhythms, theta activity has been strongly associated with attentional control, working memory, and internal cognitive processing (13). Frontal theta oscillations are particularly linked to cognitive control processes, including performance monitoring and conflict resolution (14). Elevated theta activity during resting-state conditions has been reported in individuals experiencing emotional dysregulation or depressive symptoms (12). In contrast, beta oscillations are generally associated with cortical activation, alertness, and active cognitive engagement (15). Increased beta activity has been linked to heightened arousal and anxiety-related states (16).

One EEG-derived index that has received considerable attention in both cognitive neuroscience and clinical research is the theta/beta ratio (TBR). The TBR reflects the relative balance between slower theta oscillations and faster beta oscillations and has been widely investigated as an indicator of attentional regulation and executive functioning (17). Early research on TBR focused primarily on attention-deficit/hyperactivity disorder (ADHD), where elevated theta activity combined with reduced beta activity was frequently observed during resting-state EEG recordings (18). Although the diagnostic specificity of TBR for ADHD has been debated, accumulating evidence suggests that this measure reflects broader neural mechanisms related to cognitive control and behavioral regulation (19).

More recently, researchers have begun to explore the association between TBR and psychological functioning in non-clinical populations. Several studies have suggested that resting-state TBR may be associated with individual differences in cognitive flexibility, attentional control, and emotional regulation (20, 21). For instance, Schutte et al. (20) reported that higher TBR values were associated with poorer reversal learning performance, indicating reduced cognitive flexibility. Similarly, Putman and Verkuil (21) found that TBR was related to reward sensitivity and emotional regulation strategies in healthy adults. These findings suggest that TBR may reflect neural processes involved in self-regulation and adaptive behavior.

Despite increasing interest in EEG-based indicators of psychological functioning, relatively few studies have examined the relationship between resting-state TBR and multidimensional psychological symptoms among college students. University populations represent a particularly relevant group for investigating such relationships because young adulthood is characterized by ongoing development of frontal brain networks involved in executive functioning and emotional regulation (22).

Another important consideration is that psychological distress in non-clinical populations often involves overlapping symptom domains rather than discrete psychiatric diagnoses. Transdiagnostic approaches to psychopathology emphasize shared neural mechanisms underlying multiple forms of psychological symptoms (23). Comprehensive measures such as the SCL-90 allow researchers to capture multidimensional patterns of psychological distress, providing a more holistic understanding of mental health status (7).

Given these considerations, investigating the association between EEG oscillatory activity and multidimensional psychological symptoms may provide valuable insights into the neural correlates of mental health vulnerability in university populations. In particular, examining the theta/beta ratio in frontal brain regions may help clarify the relationship between neural oscillatory balance and psychological functioning.

Therefore, the present study aimed to investigate whether resting-state frontal theta/beta ratio is associated with psychological symptom severity among college students. Resting-state EEG recordings were obtained from undergraduate participants during an eyes-closed condition, and spectral power in the theta (4–7 Hz) and beta (14–30 Hz) frequency bands was extracted from frontal electrodes (F3 and F4). Psychological symptoms were assessed using the SCL-90. It was hypothesized that students with higher psychological symptom severity would exhibit greater frontal theta activity, increased beta activity, and elevated theta/beta ratio compared with students reporting lower levels of psychological symptoms.

## Methods

### Participants

A total of 222 undergraduate students were recruited from a comprehensive university through campus advertisements and classroom announcements. Participants were aged between 18 and 25 years and were enrolled as full-time students at the time of the study. All participants provided written informed consent prior to participation. The study protocol was approved by the Institutional Review Board of the university and was conducted in accordance with the principles of the Declaration of Helsinki.

All participants were instructed to avoid caffeine, nicotine, and intense exercise for at least 2 hours before recording. They were screened to ensure that they met the inclusion criteria. Inclusion criteria were: (1) being a currently enrolled undergraduate student, (2) age between 18 and 25 years, and (3) willingness to participate in EEG recording and psychological assessment. Exclusion criteria included: (1) a self-reported history of neurological disorders, (2) current psychiatric diagnosis or ongoing psychiatric treatment, (3) use of psychoactive medications that could influence EEG activity, and (4) excessive alcohol or substance use. All participants completed the Symptom Checklist-90 (SCL-90) to assess psychological symptoms. According to the China norm of the SCI-90 total score, 160 points is the clinical threshold, and scores above 160 may indicate the presence of certain psychological disorders (24). Based on this, the subjects in this study were divided into a high-score group (> 160; 130 participants) and a low-score group (≤ 160; 92 participants). Demographic information including age, gender, and academic major was also collected through a self-report questionnaire.

### Psychological assessment

Psychological symptoms were assessed using the Symptom Checklist-90 (SCL-90), a widely used self-report instrument designed to evaluate a broad range of psychological problems and symptoms of psychopathology. The SCL-90 consists of 90 items that measure nine primary symptom dimensions: somatization, obsessive–compulsive symptoms, interpersonal sensitivity, depression, anxiety, hostility, phobic anxiety, paranoid ideation, and psychoticism.

Participants were asked to rate the extent to which each symptom had distressed or bothered them during the past week on a five-point Likert scale ranging from 1 (not at all) to 5 (extremely). The instrument provides both individual dimension scores and a Global Severity Index (GSI), which reflects overall psychological distress.

The SCL-90 has demonstrated good reliability and validity in both clinical and non-clinical populations and has been widely used in studies examining psychological health among university students. In the present study, the total SCL-90 score was used as an indicator of overall psychological symptom severity. Higher scores indicate greater levels of psychological distress.

### EEG recording procedure

Resting-state electroencephalography (EEG) was recorded in a quiet, electrically shielded laboratory environment. Participants were seated comfortably in a chair and were instructed to remain relaxed and minimize movement during the recording session.

EEG signals were recorded using a two-channel EEG system with electrodes positioned according to the international 10–20 system (Chutzpah Digital Intelligence Technology Co. Ltd, Ningbo, China). For the purposes of the present study, analyses focused on frontal electrodes F3 and F4, which are commonly associated with cognitive control and executive functioning processes (13).

Before recording, electrode impedances were maintained below 5 kΩ to ensure high-quality signal acquisition. EEG signals were sampled at an appropriate sampling rate of 250 Hz and were referenced to linked mastoids during recording.

Resting-state EEG was recorded under an eyes-closed condition for approximately 5 minutes. Participants were instructed to close their eyes, remain awake, and avoid excessive movement or muscle tension during the recording period. Eyes-closed resting-state conditions are commonly used in EEG research to minimize visual input and capture spontaneous neural activity. During recording, we monitored participants in real time through an observation window to ensure wakefulness (In addition, post-recording, EEG segments with prominent alpha-dominant slow-wave patterns (indicative of drowsiness) were excluded).

### EEG data pre-processing

EEG data preprocessing was conducted using standard procedures for artifact removal and signal cleaning. Raw EEG data were first visually inspected to identify segments containing excessive noise or recording artifacts. A band-pass filter was applied to retain frequencies within the typical EEG range (1–40 Hz). Artifacts related to eye movements, muscle activity, and other physiological noise were identified and removed using a combination of automatic and manual artifact rejection procedures. Artifact rejection was performed using an amplitude threshold of ±75 μV; epochs exceeding this threshold were automatically rejected, followed by manual verification to retain only clean segments. Segments containing large amplitude fluctuations or movement artifacts were excluded from further analysis.

Only artifact-free EEG segments were retained for spectral analysis. Specifically, 2-second non-overlapping artifact-free epochs were extracted, with approximately 100–120 valid segments per participant included for subsequent FFT computation.These procedures ensured that the EEG signals used in subsequent analyses accurately reflected neural activity rather than external noise or physiological artifacts.

### Spectral analysis and theta/beta ratio calculation

Spectral analysis of EEG data was conducted to estimate power within specific frequency bands. Power spectral density was calculated using fast Fourier transform (FFT) methods performed in MATLAB R2021b applied to artifact-free EEG segments. A Hamming window was used for FFT computation; 2-second non-overlapping epochs were analyzed, with a frequency resolution of 0.5 Hz.

Two primary frequency bands were analyzed in the present study: Theta band (4–7 Hz) and Beta band (14–30 Hz). Absolute spectral power values were extracted for each frequency band from the frontal electrodes F3 and F4. The theta/beta ratio (TBR) was then calculated by dividing theta power by beta power for each electrode site.

The TBR is considered an index reflecting the balance between slower and faster neural oscillations and has been widely used in studies examining attention regulation and cognitive control processes. For each participant, the average TBR value across frontal electrodes was computed and used in subsequent statistical analyses.

### Statistical analysis

All statistical analyses were conducted using SPSS statistical software (version 28.0). A two-way repeated-measures analysis of variance (ANOVA) was performed to examine group differences in theta and beta power as well as TBR, respectively, with Electrode (F3, F4) as the within-subject factor and Group (high symptom vs. low symptom) as the between-subject factor. In addition, Pearson correlation analyses were performed to examine the associations between EEG measures (theta and beta power, TBR) and SCL-90 scores, including both total scores and individual symptom dimensions. Bonferroni correction was applied to control Type I error, and adjusted p-values are reported. Effect sizes were reported as partial eta squared (η²) for ANOVA. The significance level for all statistical tests was set at p <.05.

## Results

### Sample characteristics

A total of 222 undergraduate students participated in the study. Participants ranged in age from 18 to 25 years (M = 20.34, SD = 1.42) and included both male and female students from various academic disciplines. No significant differences were observed between the two groups in terms of age or gender distribution (all p >.05), indicating that demographic characteristics were comparable across groups.

Descriptive statistics for demographic variables and psychological symptom scores are presented in **Table 1**. The mean total score of the Symptom Checklist-90 (SCL-90) was 183.8 (SD = 64.4), indicating a broad range of psychological symptom severity within the sample.

**Table 1:** Descriptive statistics of SCL-90 scores by group (M ± SD).

SCL-90 scores	Group*	
	Low-scores	High-scores
Depression	1.33 ± 0.33	2.77 ± 0.61
Anxiety	1.29 ± 0.28	2.62 ± 0.63
Hostility	1.27 ± 0.33	2.36 ± 0.69
Interpersonal sensitivity	1.42 ± 0.41	2.79 ± 0.60
Somatization	1.19 ± 0.24	2.16 ± 0.65
Psychoticism	1.27 ± 0.31	2.45 ± 0.56
Compulsive	1.64 ± 0.48	3.09 ± 0.63
Terror	1.19 ± 0.23	2.03 ± 0.67
Paranoid	1.32 ± 0.37	2.46 ± 0.66
Others	1.30 ± 0.33	2.52 ± 0.61
Total score	119.5 ± 23.3	229.4 ± 40.8

*, ps < 0.001, group comparison of each dimension and total scores.

For group comparison analyses, participants were categorized into two groups based on the SCL-90 total scores, with 160 points as the clinical threshold (24). The high-score group included 130 participants with scores ranging from 162 to 341 (M = 229.4, SD = 40.8), whereas the low-score group included 92 participants with scores ranging from 90 to 160 (M = 119.5, SD = 23.3). An independent-samples t-test confirmed that the high-score group exhibited significantly higher SCL-90 total scores than the low-score group, t(220) = 23.28, p <.001, d = 3.19.

### Group differences in EEG spectral power

Mean values of EEG spectral measures for each group are illustrated in **Figure 1**.

**Figure 1:**
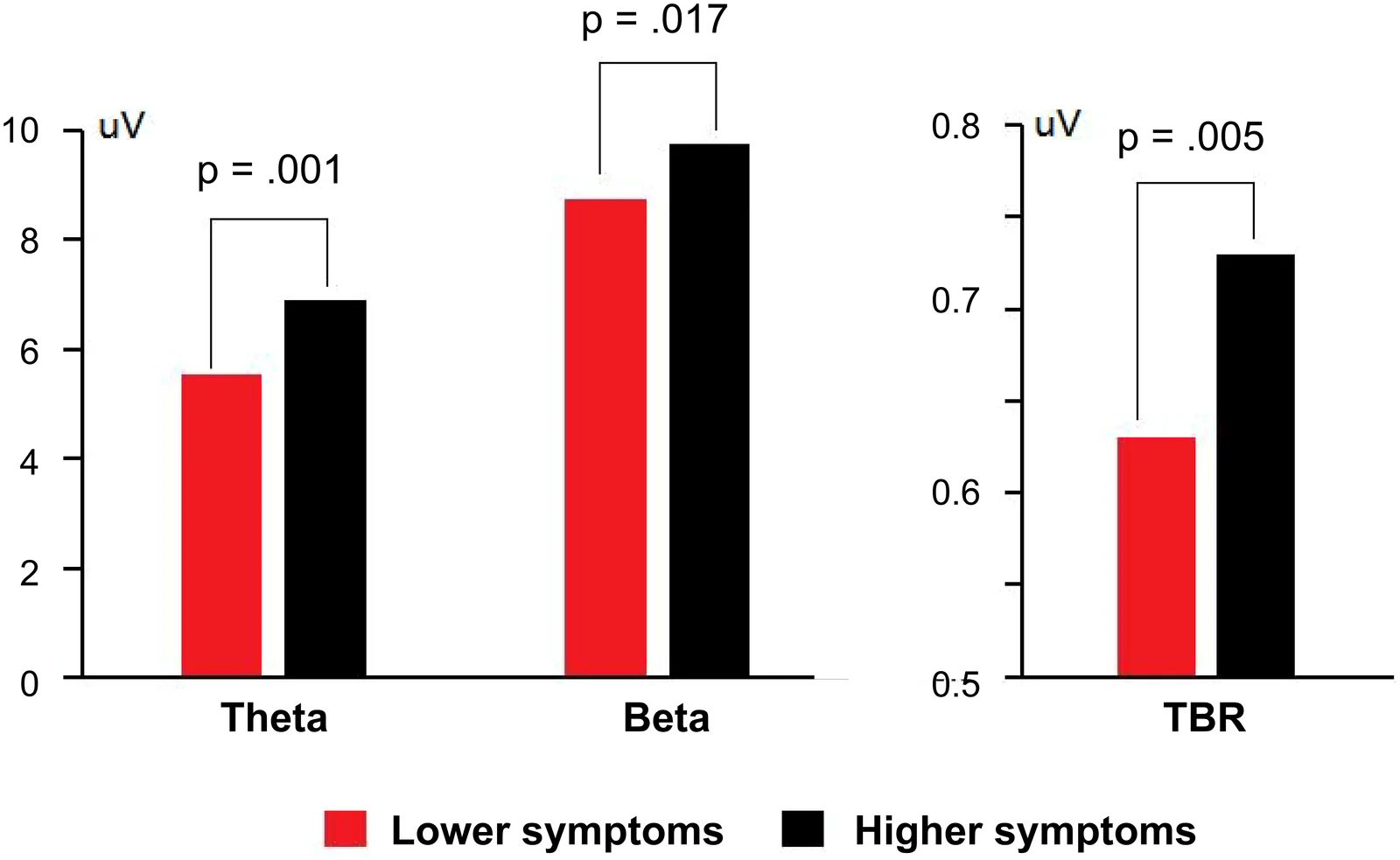
EEG theta/beta analysis illustration in two groups.

For theta band activity, a two-way repeated-measures ANOVA revealed a significant main effect of Group, indicating that participants in the high-score group exhibited greater frontal theta power (M = 6.9 μV) than those in the low-score group (M = 5.5 μV), F(1, 220) = 12.25, p <.001, ηp² = .059.The main effect of Electrode was not significant (F < 1). However, a significant Group × Electrode interaction was observed, F(1, 220) = 5.50, p = .02, ηp² = .024. Follow-up analyses indicated a significant asymmetry in the low-score group, with theta power slightly higher at F4 (5.7 μV) than at F3 (5.4 μV), p = .05. In contrast, no significant asymmetry was observed in the high-score group (F3 = 7.0 μV, F4 = 6.8 μV; p = .197).

For beta band activity, the ANOVA revealed a significant main effect of Group, with higher frontal beta power observed in the high-score group (M = 9.8 μV) compared with the low-score group (M = 8.7 μV), F(1, 220) = 5.74, p = .017, ηp² = .025. The main effect of Electrode was not significant, F(1, 220) = 3.00, p = .085, ηp² = .013. However, a significant Group × Electrode interaction was detected, F(1, 220) = 7.26, p = .008, ηp² = .032. Further analyses showed that the group difference was evident at the left frontal electrode (F3) (p <.001), but not at the right frontal electrode (F4) (p = .38). Additionally, a significant asymmetry was observed in the low-score group, with beta power higher at F4 (9.2 μV) than at F3 (8.3 μV), p = .004. No significant asymmetry was observed in the high-score group (F3 = 9.9 μV, F4 = 9.7 μV; p = .456).

For the theta/beta ratio, the two-way ANOVA revealed a significant main effect of Group, indicating that participants in the high-score group exhibited a significantly greater frontal TBR (M = 0.70) compared with those in the low-score group (M = 0.63), F(1, 220) = 8.17, p = .005, ηp² = .036. Neither the main effect of Electrode nor the Group × Electrode interaction reached statistical significance (both F < 1).

### Correlations between EEG measures and psychological symptoms

Pearson correlation analyses were conducted to examine the relationships between EEG measures and psychological symptom severity. Correlation coefficients with Bonferroni correction between EEG measures and SCL-90 symptom dimensions are presented in **Table 2**.

**Table 2:** Correlation between EEG measures and psychological symptoms.

SCL-90 scores		Theta	Beta	TBR
SCL-90-Somatization	r	.210^**^	.117	.168^*^
	p	.002	.083	.012
SCL-90-Compulsive	r	.281^**^	.134^*^	.276^**^
	p	.000	.045	.000
SCL-90-Interpersonal sensitivity	r	.260^**^	.162^*^	.212^**^
	p	.000	.015	.002
SCL-90-Depression	r	.248^**^	.149^*^	.206^**^
	p	.000	.027	.002
SCL-90-Anxiety	r	.270^**^	.199^**^	.191^**^
	p	.000	.003	.004
SCL-90-Hostility	r	.198^**^	.105	.167^*^
	p	.003	.119	.013
SCL-90-Fear	r	.197^**^	.126	.149^*^
	p	.003	.061	.026
SCL-90-Paranoia	r	.217^**^	.109	.193^**^
	p	.001	.104	.004
SCL-90-Psychoticism	r	.276^**^	.150^*^	.232^**^
	p	.000	.025	.000
SCL-90-Others	r	.277^**^	.195^**^	.199^**^
	p	.000	.004	.003
SCL-90-Total	r	.278^**^	.165^*^	.229^**^
	p	.000	.014	.001

*, p <.05; **, p <.01.

Frontal theta power was positively correlated with the total SCL-90 score, r = .278, p <.001. Additionally, theta power was significantly associated with all SCL-90 symptom domains (all r >.197, p <.003), indicating that greater theta activity was related to higher levels of psychological distress.

Similarly, beta power showed a significant positive correlation with the total SCL-90 score, r = .165, p = .014. Further analyses revealed that beta power was positively associated with several symptom domains, including depression, anxiety, hostility, interpersonal sensitivity, and psychoticism (all p <.05), whereas no significant associations were observed for the remaining symptom domains (all p >.05).

Importantly, the theta/beta ratio demonstrated a significant positive correlation with overall psychological symptom severity, r = .229, p = .001. Additional analyses indicated that TBR was positively correlated with all SCL-90 symptom dimensions (all r >.14, p <.03).

Taken together, these findings suggest that elevated resting-state frontal theta/beta ratio may be associated with broader patterns of psychological distress in college students.

## Discussion

The present study examined the association between resting-state frontal theta/beta ratio (TBR) and psychological symptoms among college students using EEG recordings and multidimensional psychological assessments. The findings revealed that students with higher levels of psychological symptoms exhibited significantly greater frontal theta power, beta power, and theta/beta ratio compared with those reporting low-score severity. Furthermore, TBR was positively correlated with overall psychological distress as measured by the SCL-90. These results provide evidence that resting-state neural oscillatory activity may be associated with psychological functioning in non-clinical university populations.

One key finding of this study is the increased frontal theta activity observed among students reporting greater psychological symptom severity. Theta oscillations have been widely linked to cognitive control and attentional regulation processes, particularly within frontal brain regions (13). Frontal theta activity is often interpreted as reflecting increased cognitive effort or engagement of executive control mechanisms during complex cognitive tasks (14). In resting-state conditions, elevated theta activity may indicate internally directed cognitive processing or inefficient neural regulation of attentional resources. Previous research has reported increased theta activity in individuals experiencing depressive symptoms and emotional dysregulation (12), suggesting that heightened theta oscillations may reflect neural mechanisms associated with psychological distress.

In addition to theta activity, the present study also observed increased beta power among students with higher psychological symptom levels. Beta oscillations are typically associated with cortical arousal, attentional engagement, and active cognitive processing (15). Elevated beta activity has been linked to anxiety-related states and heightened emotional reactivity (16). In the context of psychological distress, increased beta power may reflect heightened physiological arousal or persistent cognitive engagement with negative emotional experiences. These findings are consistent with previous EEG research demonstrating that individuals experiencing anxiety and stress-related symptoms often exhibit increased beta oscillatory activity during resting-state conditions (11).

A central contribution of the present study is the identification of a significant association between the theta/beta ratio and multidimensional psychological symptoms. Importantly, the pattern of TBR observed in the present study differs from the classical ADHD model (17). In ADHD, elevated TBR typically arises from increased theta and decreased beta activity. In contrast, our findings revealed simultaneous increases in both theta and beta power in the high-symptom group. Therefore, TBR should be interpreted as a composite index of relative oscillatory balance between slow and fast neural oscillations, rather than a direct or specific biomarker of cognitive control. The ratio reflects the relative dynamic between theta and beta oscillations, but does not carry the same neurophysiological meaning as in previous ADHD literature. Caution is warranted in overinterpreting TBR as a marker of cognitive control efficiency, as the ratio is highly sensitive to relative scaling between the two frequency bands and may obscure independent changes in theta and beta power (also see, 19).

The positive association between TBR and psychological symptom severity observed in the present study suggests that individuals experiencing greater psychological distress may exhibit altered neural oscillatory balance in frontal brain regions. Previous studies have reported that TBR may be related to cognitive flexibility, reward processing, and behavioral adaptation (20). Additionally, Putman and Verkuil (21) demonstrated that resting-state TBR is associated with emotional regulation strategies and sensitivity to environmental feedback. These findings support the interpretation that TBR may reflect neural processes involved in self-regulation and adaptive behavior (21).

The observed associations between TBR and multiple SCL-90 symptom dimensions further suggest that this neural measure may reflect general psychological vulnerability rather than disorder-specific mechanisms. Psychological symptoms in non-clinical populations frequently occur in overlapping patterns rather than discrete diagnostic categories. Transdiagnostic perspectives in psychopathology emphasize shared neural circuits underlying various forms of emotional distress (23). From this perspective, altered oscillatory balance between theta and beta rhythms may represent a neural signature of broader dysregulation within cognitive and emotional control systems.

The frontal cortex plays a critical role in executive functioning, decision-making, and emotional regulation (25). Neural oscillations recorded from frontal electrodes such as F3 and F4 are closely associated with cognitive control processes. Frontal theta oscillations are particularly involved in performance monitoring and conflict detection (13), whereas beta oscillations are associated with sustained attention and cortical activation (16). The present findings suggest that alterations in the balance between these oscillatory rhythms may reflect disruptions in neural systems responsible for regulating attention and emotion.

These findings also have important implications for mental health monitoring in university populations. The prevalence of psychological distress among college students has increased substantially in recent years (1, 2). Early identification of individuals at risk for mental health difficulties is therefore an important priority for public health and educational institutions. While psychological assessments are typically based on self-report measures, objective neurophysiological indicators such as EEG may provide additional insights into neural processes associated with mental health vulnerability. Resting-state EEG measures such as the theta/beta ratio may therefore contribute to the development of complementary approaches for assessing psychological risk.

Several limitations of the present study should be considered. First, the cross-sectional design limits the ability to draw causal conclusions regarding the relationship between neural oscillatory activity and psychological symptoms. Longitudinal studies are needed to determine whether alterations in TBR precede the development of psychological distress or emerge as a consequence of existing symptoms. Second, psychological symptoms were assessed using self-report measures, which may be influenced by response biases or subjective interpretations. Future research could incorporate clinical diagnostic interviews or behavioral assessments to provide more comprehensive measures of psychological functioning. Third, the present study focused primarily on frontal EEG electrodes. Although frontal regions play a central role in cognitive control processes, future studies could examine broader neural networks involving additional cortical regions. In addition, longitudinal studies may help clarify whether resting-state EEG markers can predict future mental health outcomes in university populations. Fourth, the present study did not control for menstrual cycle phase in female participants. Hormonal fluctuations across the menstrual cycle are known to modulate resting EEG oscillations, which may have introduced residual variance and potentially obscured subtle sex-related effects. Future studies should consider controlling for menstrual cycle phase to reduce potential confounding. Fifth, multiple correlation analyses were conducted across SCL-90 dimensions; although Bonferroni correction was applied, the risk of Type I error cannot be completely eliminated, which should be noted when interpreting the results.

In summary, the present study provides evidence that resting-state frontal theta/beta ratio is associated with psychological symptom severity among college students. Students reporting higher levels of psychological distress exhibited greater frontal theta power, beta power, and TBR compared with students reporting low-score levels. These findings suggest that frontal TBR may serve as a candidate neurophysiological correlate associated with mental health in non-clinical populations. Investigating EEG-based markers of psychological functioning may contribute to the development of more objective approaches to mental health assessment and early detection in university populations.

## Data Availability

The raw data supporting the conclusions of this article will be made available by the authors, without undue reservation.
